# The effect and safety of ropinirole in the treatment of Parkinson disease: A systematic review and meta-analysis

**DOI:** 10.1097/MD.0000000000027653

**Published:** 2021-11-19

**Authors:** Jiali Zhu, Min Chen

**Affiliations:** Department of Neurology, Affiliated Hospital of Guilin Medical University, China.

**Keywords:** meta-analysis, Parkinson disease, RCT, ropinirole, safety, treatment

## Abstract

**Background:**

It is necessary to conduct a meta-analysis of the clinical randomized controlled trials (RCTs) on ropinirole in the treatment of Parkinson disease (PD), to explore the effects and safety of ropinirole, and to provide a theoretical basis for clinically safe and rational drug use.

**Methods:**

RCTs on the effectiveness and safety of ropinirole in the treatment of PD were searched. We searched Dutch medical literature database, Pubmed, Cochrane Library, China National Knowledge Infrastructure, Wanfang Knowledge Service Platform up to December 15, 2020. The Cochrane risk bias assessment tool was used to evaluate the quality of the included literature, and the RevMan5.3 software was used for meta-analysis.

**Results:**

A total of 12 RCTs with 3341 patients were included. The changes of Parkinson Disease Rating Scale Part II score (mean difference = –2.23, 95% confidence interval [CI] –2.82 to –1.64) and Parkinson Disease Rating Scale Part III scores (mean difference = –4.93, 95%CI –5.25 to –4.61) in the ropinirole group was significantly lower than that in the control group. The incidence of dizziness (odd risk [OR] = 1.85, 95%CI 1.50–2.28), nausea (OR = 2.17, 95%CI 1.81–2.59), vomiting (OR = 2.73, 95%CI 1.47–5.09), and lethargy (OR = 2.19, 95%CI 1.39–3.44) in the ropinirole group was significantly higher than that in the control group (all *P* < .05), and there were no significant differences in the incidence of headache (OR = 1.14, 95%CI 0.79–1.65) and insomnia (OR = 1.06, 95%CI 0.72–1.55) were found between 2 groups (all *P* > .05).

**Conclusions:**

Ropinirole can help improve the ability of daily living and exercise function of PD patients, but it will increase the incidence of related adverse reactions, which needs to be further confirmed by subsequent large-scale, high-quality RCTs.

## 1 Introduction

Parkinson disease (PD) is a common neurodegenerative disease in middle-aged and elderly people.^[1]^ Its symptoms include typical motor symptoms and non-motor symptoms. At present, early and mid-term PD is still dominated by drug therapy.^[2]^ Dopamine agonist (DA) has been widely used in the early monotherapy of PD and the combination therapy with levodopa in the middle and late stages.^[3]^ Although there is no more recognized evidence that one type of DA is better than another type of DA, ergot DA is no longer used as the first-line treatment for PD due to its fibrotic side effects.^[4]^ However, non-ergot DA continues to be used as the first-line treatment for PD.^[5]^ At present, new long-acting non-ergot DA preparations such as ropinirole have been developed, and their effectiveness and safety have been extensively studied to guide the clinical drug use and treatment of PD.

In the past, dopamine receptor agonists were mostly partial agonists of the receptor. At present, the non-ergot receptor agonists pramipexole and piribedil hydrochloride are widely used at home and abroad.^[6]^ Ropinirole as a new generation of non-ergot alkaloid selective dopamine D2/D3 receptor agonists, it was first marketed in the UK in 1996 and was approved by the Food and Drug Administration for PD treatment in 1998. It has a unique pharmacological effect and a long half-life, it can last for a long time on dopamine receptors, and it is beneficial to reduce the number of medications and drug dosage.^[7]^ Understanding the effectiveness and safety of ropinirole in the treatment of PD has important guiding significance for clinical medication. Previous studies^[8,9]^ have focused on the role of ropinirole in PD, yet the results remained inconsistent. Therefore, we aimed to conduct a meta-analysis to investigate the effect and safety of ropinirole in the treatment of PD, to provide insights to the clinical treatment of PD.

## 2 Methods

### 2.1 Ethical consideration

Ethical approval and patient informed consent were not necessary since our study was a meta-analysis and systematic review.

### 2.2 Literature search

We used computers to search the Dutch medical literature database (Embase), the U.S. National Library of Medicine Medical Literature Retrieval System (Pubmed), the Cochrane Library, China National Knowledge Infrastructure (CNKI), Wanfang Knowledge Service Platform for informal non-inferiority design studies on the effect and safety of ropinirole in the treatment of PD. At the same time, we manually searched related documents and references. The search deadline was December 15, 2020. The database search term used was: (“Ropinirole” OR “non-ergot dopamine agonist” OR “NEDA”) AND (“Parkinson's Disease” OR “PD”). Two authors independently conducted literature search and screening.

### 2.3 Inclusion and exclusion criteria

The inclusion criteria of this meta-analysis were as follows: All participants were not limited in gender, age, and nationality, and the diagnosis of PD met the relevant PD diagnostic criteria. The interventions should compare the ropinirole and control treatments. The study design is a randomized controlled trial (RCT). Exclusion criteria: Non-RCT research design. Patients with a history of brain stereotactic surgery in the patient's medical history, or patients with serious underlying diseases and mental disorders. The study sample was unclear or the relevant outcome data were incomplete.

### 2.4 Quality evaluation

Two evaluators independently completed the data extraction and quality evaluation, and then checked and compared each other. If the opinions were inconsistent, they would discuss with the third evaluator. Cochrane collaboration's tool^[10]^ for assessing risk of bias was used for quality evaluation, and each item was divided into “low bias”, “unclear”, and “high bias”. “Low bias” means that there is no risk of bias, which is indicated by a green area on the Cochrane evaluation scale; “unclear” means that the evaluator cannot judge whether there is a bias, and it is indicated by a yellow area on the Cochrane evaluation scale; “highly biased” indicates that there is a risk of bias, which is indicated by a red area on the Cochrane evaluation scale.

### 2.5 Data extraction

We extracted the number of cases, gender ratio, average age, Hoehn-Yahr scale, treatment dose, and course of treatment in each RCT. The extracted outcome indicators included: the change in the total activity of daily living score in the Parkinson Disease Rating Scale Part II (UPDRS II) from the baseline; the change in the total motor function test score in Part III (UPDRS III) from the baseline, and the incidence of adverse events after treatment with ropinirole, such as dizziness, nausea, vomiting, drowsiness, insomnia, hallucinations, dyskinesia.

### 2.6 Statistical analysis

We used RevMan5.3 statistical software for meta-analysis. Continuous variables use mean difference (MD), and binary variables use odd risk (OR) as the statistic used for efficacy analysis, with 95% confidence interval (CI) represented each effect size. The heterogeneity of the data was tested by I^2^ statistic. In this study, the random effects model was used to calculate the total results. According to the possible heterogeneity factors, subgroup analysis and sensitivity analysis were performed to clarify the reasons for the heterogeneity. *P* < .05 indicated that the difference between groups was statistically significant.

## 3 Results

### 3.1 Study selection

**Figure 1:**
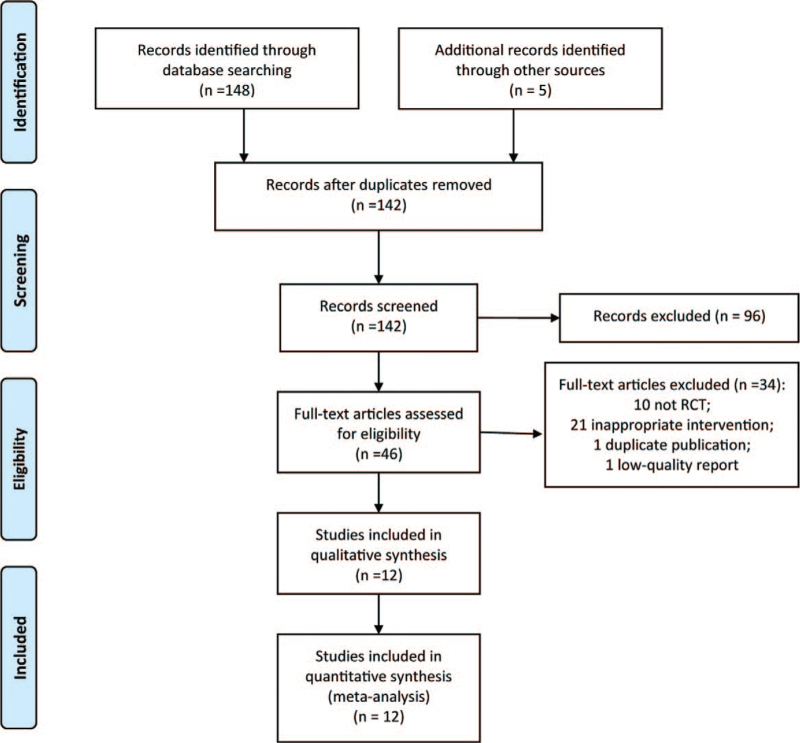
The flow chart of study selections.

Through the initial database search, 142 potential documents were initially obtained. With reference to the inclusion and exclusion criteria, 96 articles were excluded by reading the title and abstract. We searched and read the full text of the remaining 46 articles, and further excluded 34 articles by reading the full text with reasons including non-RCT study design, different intervention methods. Three associated RCTs^[11–13]^ were excluded because they compared the ropinirole and other drugs, which failed to meet the inclusion criteria of this meta-analyses. A total of 12 RCTs^[14–25]^ with 3341 patients were included finally, including 1855 patients in the ropinirole group and 1486 patients in the control group. The flow chart of study selections was presented in Figure 1.

### 3.2 General characteristics of included RCTs

Among the 12 included studies, 1 study^[25]^ was a superior trial design, 7 studies^[14–20]^ were a non-inferiority trial design, and 4 studies^[21–24]^ were an informal non-inferiority design. Six RCTs^[14,17,18,22–24]^ were conducted in USA, 1 in Italy,^[15]^ Britain,^[16]^ Israel,^[17]^ Japan,^[19]^ France,^[21]^ and China,^[25]^ respectively. The doses of ropinirole and the duration of treatment were different in different included studies. UPDRS score II & III were not used in all included studies. The characteristics of included RCTs were presented in Table 1.

**Table 1:** The characteristics of included patients.

Studies	Country	Sample size	Dose (mg/d)	Duration of treatment (wk)
Adler 1997	USA	241	0.75–24.0	24
Barone 2007	Italy	624	0.75–24.0	40
Brooks 1998	Britain	63	1. 0–10. 0	12
Giladi 2007	Israel	346	0.5–24.0	37
Lieberman 1998	USA	149	0.75–24.0	24
Mizuno 2007	Japan	241	0.75–15.0	16
Pahua 2007	USA	391	2.0–24.0	24
Rascol 1996	France	46	1. 0–8. 0	12
Scthi 1998	USA	147	3.0–24.0	48
Singer 2007	USA	398	0.75–24.0	40
Zesiewiez 2017	USA	350	4.0–24.0	17
Zhang 2013	China	345	2.0–24.0	24

### 3.3 Quality evaluation of included studies

**Figure 2:**
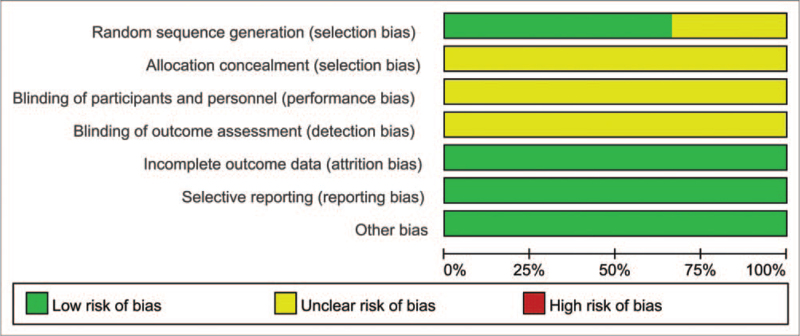
Risk of bias graph.

**Figure 3:**
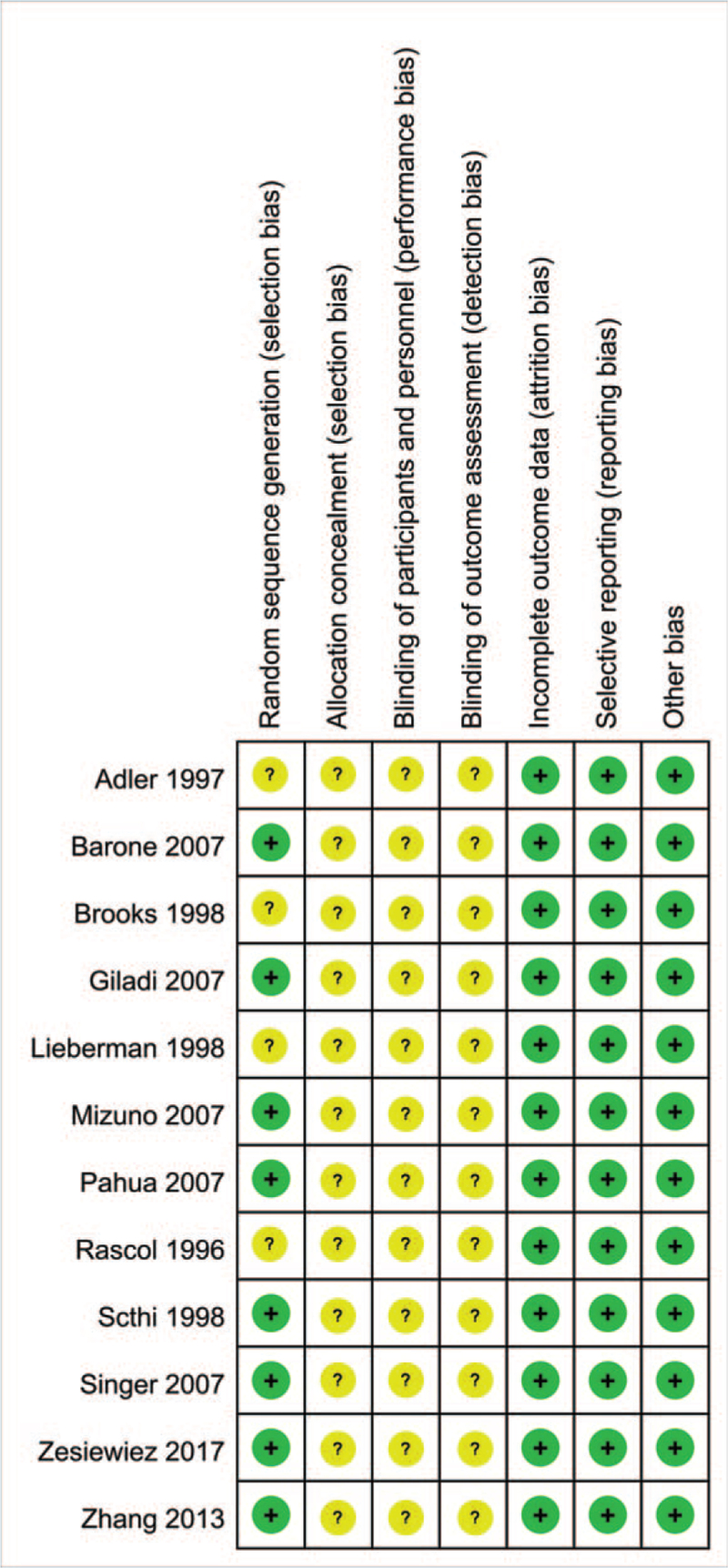
Risk of bias summary.

We evaluated the quality of all the included literature according to the research quality evaluation criteria recommended by Cochrane Handbook 5.1.0. All included studies were RCTs. Four studies^[14,16,19,21]^ only mentioned randomization without specifying specific methods. The remaining studies^[15,17,18,20,22–25]^ all described specific methods. None of the studies explained the hiding of allocation, and none of the studies explained the blinding method. In terms of the completeness of the result data, all studies have no missing data. None of the 12 RCTs reported selective results and other sources of bias. The quality evaluation of the included studies was shown in Figures 2 and 3.

### 3.4 Meta-analyses

#### 3.4.1 Changes of UPDRS II score

**Figure 4:**
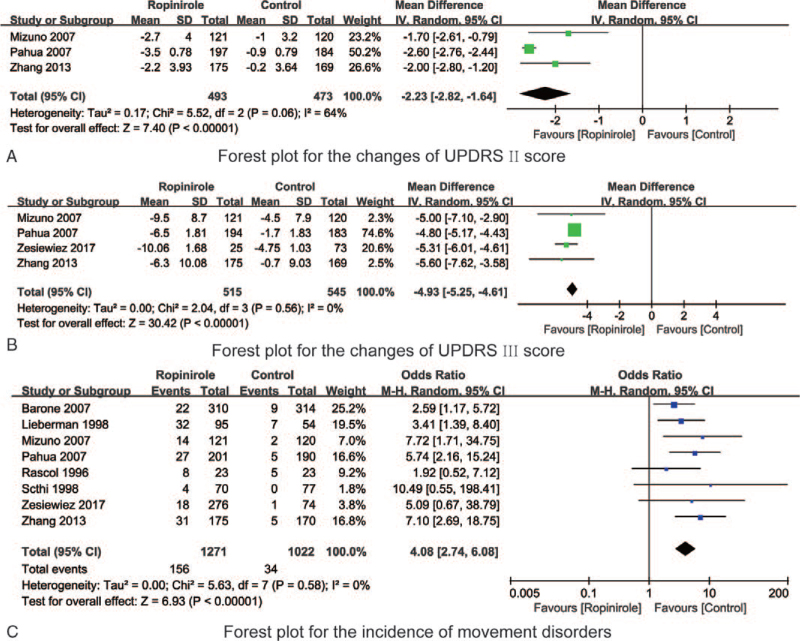
The forest plots for synthesized outcomes.

Three RCTs^[19,20,25]^ reported the changes of UPDRS II scores before and after treatment with ropinirole or control in PD patients. The heterogeneity test indicated that the synthesized results of the various studies have moderate heterogeneity (*P* = .06, I^2^ = 64%). Meta-analysis results showed that the changes of UPDRS II score in the ropinirole group was significantly lower than that in the control group (MD = –2.23, 95%CI –2.82 to –1.64) (see Fig. 4A).

#### 3.4.2 Changes of UPDRS III score

Four RCTs^[19,20,24,25]^ reported the changes of UPDRS III scores before and after treatment with ropinirole or control in PD patients. The heterogeneity test indicated that the synthesized results of the various studies have no heterogeneity (*P* = .56, I^2^ = 0%). Meta-analysis results showed that the changes of UPDRS III score in the ropinirole group was significantly lower than that in the control group (MD = –4.93, 95%CI –5.25 to –4.61) (see Fig. 4B).

#### 3.4.3 The incidence of movement disorders

Eight RCTs^[15,18–22,24,25]^ reported the incidence of movement disorders with ropinirole or control in PD patients. The heterogeneity test indicated that the synthesized results of the various studies have no heterogeneity (*P* = .58, I^2^ = 0%). Meta-analysis results showed that incidence of movement disorders in the ropinirole group was significantly lower than that in the control group (OR = 4.08, 95%CI 2.74 to –6.08) (see Fig. 4C).

#### 3.4.4 Complications

As presented in Table 2, the incidence of dizziness (OR = 1.85, 95%CI 1.50–2.28), nausea (OR = 2.17, 95%CI 1.81–2.59), vomiting (OR = 2.73, 95%CI 1.47–5.09), and lethargy (OR = 2.19, 95%CI 1.39–3.44) in the ropinirole group was significantly higher than that in the control group (all *P* < .05), and there were no significant differences in the incidence of headache (OR = 1.14, 95%CI 0.79–1.65) and insomnia (OR = 1.06, 95%CI 0.72–1.55) were found between 2 groups (all *P* > .05).

**Table 2:** The meta-analyses on the related complications between 2 groups.

Variables	Number of included RCTs	Heterogeneity (I^2^)	OR	95%CI	*P*
The incidence of dizziness	12	23%	1. 85	1.50–2.28	<.001
Incidence of nausea	12	49%	2.17	1.81–2.59	<.001
The incidence of vomiting	6	0%	2.73	1.47–5.09	.001
Incidence of headache	6	6%	1.14	0.79–1.65	.493
Incidence of lethargy	12	71%	2.19	1.39–3.44	<.001
Incidence of insomnia	7	5%	1. 06	0.72–1.55	.171

CI = confidence interval, OR = odd risk, RCT = randomized controlled trial.

## 4 Discussions

Drug treatment can improve the symptoms of PD and improve the quality of life of patients.^[26]^ At present, compound levodopa, dopamine receptor agonists, monoamine oxidase B inhibitors, catecholamine-O-methyltransferase inhibitors, etc are common drugs for the treatment of PD.^[27]^ Dopamine receptor agonists can directly act on postsynaptic dopamine receptors to improve symptoms.^[28]^ Ropinirole is a new type of dopamine D2 receptor agonist.^[29]^ A number of clinical studies^[30–32]^ have discussed its therapeutic effect and safety, but the results are not consistent. Previous meta-analysis^[33]^ has included 12 RCT studies prior to 2010 involving ropinirole, demonstrating a higher incidence of adverse event of ropinirole such as somnolence, dyskinesia in addition to dizziness, nausea, vomiting observed in this study, which may be associated to the fact that the adverse effects of ropinirole are reduced with the development of biopharmaceutical technology, this study mainly focused on the adverse effects of ropinirole, we have both focused on the therapeutic effects and safety of ropinirole in the treatment of PD. The results of this meta-analysis show that ropinirole has a significant effect in improving PD motor function and ability of daily living, but its risk of dizziness, nausea, vomiting, and lethargy is also significantly higher.

Ropinirole is a non-ergot dopamine receptor agonist that is selective for D2 and D3 dopamine receptors.^[34]^ It has negligible affinity for a wide range of central non-dopaminergic receptors, including a and β adrenergic receptors, serotonin receptor type 1, serotonin receptor type 2, benzodiazepines, and γ-GABA receptor.^[35]^ In PD patients with exercise fluctuations, ropinirole, as an adjunct to L-DA, has been proven in early trials to improve the symptoms of PD.^[36]^ It has been reported that the use of ropinirole as an adjuvant therapy can also significantly reduce the dosage of L-DA.^[37]^ UPDRS score is a scale that evaluates the severity of PD. It combines the subjective and objective perspectives of patients for a more detailed assessment from various aspects such as different motor symptoms, non-motor symptoms, and motor complications.

The safety of Ropinirole in the treatment of PD deserves further consideration. Dopamine receptor agonists have been used as anti-PD drugs since 1974, and they offer several theoretical advantages over levodopa therapy.^[38]^ Firstly, they directly stimulate dopaminergic receptors in the postsynaptic striatum, without having to pass through a degraded pool of black striatal neurons or be regulated by reduced striatal terminals to convert to dopamine.^[39]^ Secondly, they can be designed to preferentially stimulate a specific subset of dopamine receptors.^[40]^ Thirdly, they have a longer half-life than levodopa and do not compete with dietary amino acids to enter the circulation and brain. Dopamine receptor agonists, as adjuvants to levodopa, have played an established role in the treatment of PD.^[41]^ However, they are not as widely used as expected from their pharmacological characteristics, which may be related to the difficulty of managing patients with combined therapy.

The results of this safety analysis have showed that the incidence of adverse events in the ropinirole group was higher than that in the control group. The incidence of adverse reactions including dizziness, nausea, vomiting, lethargy, hallucinations, dyskinesias, fatigue were significantly higher than that of control group. In the previous reports,^[42,43]^ the incidence of ropinirole dizziness was 6% to 40%, which was related to the dosage. Dizziness is a common neurological adverse reaction in the ropinirole group in this study. At present, there is no reports on the mechanism of dizziness after ropinirole treatment. Studies^[44,45]^ have reported the incidence of insomnia is 6% to 26%. The mechanism of sleep disorders may be related to adverse dopaminergic reactions. In animal models, D2 receptors have a dual role. Low doses stimulate presynaptic receptors to produce a sedative effect, and high doses stimulate postsynaptic receptors to promote wakefulness, low-dose dopamine can cause sleepiness in PD patients, and high-dose dopamine can cause insomnia.^[46]^ In addition, studies have reported that orthostatic hypotension is very common in PD patients. It has been reported that the incidence of inpatients with PD is 43% to 58%, and the incidence of PD patients in the community is 47%.^[47]^ Compound levodopa and dopamine receptor agonists can cause orthostatic hypotension.^[33]^ Studies^[48,49]^ have found that dopamine receptor agonists may cause insufficient increase in norepinephrine secretion when the position is changed, and then cause orthostatic hypotension. In addition, some research results^[50,51]^ suggest that ropinirole has a certain substitute value for patients with severe headache, insomnia, orthostatic hypotension, constipation, and other symptoms caused by long-term levodopa, and further clinical research is needed in this regard.

This study still has certain limitations that must be considered. Firstly, the number of reports retrieved in this study was small and the sample size was not large, which did not fully represent the efficacy and safety of ropinirole in the treatment of PD. And the incidence of movement disorders are heterogeneous so that the effects and side effects were different, more studies on the safety of ropinirole are needed in the future. Secondly, due to the incomplete data of some reports, many useful data could not be extracted, and most of the data were from European and American countries, and there were few data from Asian countries, which may cause bias in the outcome. Besides, we only included RCTs comparing the ropinirole vs placebo in this meta-analysis. More network meta-analyses are needed in the future to evaluate standard ropinirole vs placebo, long-acting ropinirole vs placebo, standard ropinirole vs specific comparator drugs, moreover, large-scale, high-quality RCTs with long-term follow-up period are needed for further role verification of ropinirole in the PD treatment.

## 5 Conclusions

As a non-ergot dopamine D2/D3 receptor agonist, ropinirole has been proven to be a monotherapy and adjuvant treatment of L-dopa to reduce the symptoms of PD. Ropinirole has shown effective symptom relief in the treatment of patients with PD and is usually well tolerated. Patients treated with ropinirole had a significant improvement in motor function, which was determined by the UPDRS score. However, ropinirole may be also complicated by several complications. Therefore, and further studies are needed to evaluate the adverse reactions and tolerance of PD patients taking ropinirole for a long time.

## Author contributions

M C designed research; J Z, M C conducted research; J Z, M C analyzed data; M C wrote the first draft of manuscript; M C had primary responsibility for final content. All authors read and approved the final manuscript.

**Conceptualization:** Min Chen.

**Data curation:** Jiali Zhu, Min Chen.

**Formal analysis:** Jiali Zhu, Min Chen.

**Investigation:** Min Chen.

**Methodology:** Jiali Zhu, Min Chen.

**Project administration:** Min Chen.

**Resources:** Jiali Zhu, Min Chen.

**Software:** Jiali Zhu, Min Chen.

**Supervision:** Min Chen.

**Visualization:** Min Chen.

**Writing – original draft:** Min Chen.
